# Chemsex among men who have sex with men (MSM) in Yangon, Myanmar: Prevalence, practices, and associated risk factors

**DOI:** 10.1371/journal.pone.0353993

**Published:** 2026-07-17

**Authors:** Naung Latt Htun, Myo-Myo Mon, Kyaw Soe Thant, Phyu Phyu Kyaw

**Affiliations:** 1 Telekyanmar Telehealth Clinic, Yangon, Myanmar; 2 Research & Development Unit, Ministry of Health, National Unity Government, Yangon, Myanmar; 3 National HIV/ AIDS Program, Ministry of Health, National Unity Government, Yangon, Myanmar; University of Technology Sydney, AUSTRALIA

## Abstract

**Introduction:**

Chemsex is the intentional use of psychoactive substances to facilitate, enhance, or prolong sexual encounters. It is notably common among men who have sex with men (MSM), contributing to high-risk sexual behaviors. However, limited research has focused on chemsex among MSM in Myanmar. This study aimed to assess the prevalence, practices, and associated risk factors of chemsex among MSM in Yangon, Myanmar.

**Materials and methods:**

Between December 2024 and January 2025, a cross-sectional online survey was conducted among MSM aged 18 and older living in Yangon. Participants were recruited through social media platforms, including Facebook and Telegram, as well as dating apps. Data were collected using the KoboCollect platform and analyzed in SPSS. Descriptive statistics summarized demographic and behavioral data, and multivariate analyses identified factors associated with chemsex.

**Results:**

The study included 415 MSM participants with a mean age of 30.1 years. The majority (71.3%) were between 18 and 34 years old. Most identified as gay (76%), had a university or postgraduate degree (62%), and 60% were employed. Approximately 26% reported engaging in chemsex at some point, with 20.7% having done so within the past six months. The most commonly reported substances were methamphetamine (69.8%), poppers (33.7%), and cannabis (19.8%). Injection and inhalation were the most common routes of administration. Chemsex sessions mostly took place in hotels (76.7%) and homes (54.7%), and for nearly half of participants, sessions lasted between 4 and 12 hours. Only 10% reported consistent condom use during chemsex, while group sex was frequent – about 40% engaged with 4–5 partners, and nearly 60% participated in threesomes. Chemsex within the last 14 days was reported by 46.5%. Factors significantly associated with chemsex included identifying as gay (aOR = 3.0, 95% CI:1.5–5.9, p = 0.001), smoking (aOR = 2.8, 95% CI:1.2–4.0, p = 0.006), participation in group sex (aOR = 4.0, 95% CI: 2.4–6.6, p < 0.001), and having multiple partners (aOR = 3.3, 95% CI:1.7–6.3, p < 0.001).

**Conclusion:**

Chemsex prevalence among MSM in Yangon is alarmingly high and associated with risky behaviors. There is an urgent need for targeted interventions that focus on education, harm reduction, and improved access to MSM-specific health services.

## Introduction

Chemsex is the intentional use of psychoactive substances to facilitate, enhance, or prolong sexual encounters. It has become a public health concern in many countries over the past decade. It is common among men who have sex with men (MSM) and is linked to behaviors that increase the transmission of HIV and other sexually transmitted infections (STIs). The most commonly used substances are crystal methamphetamine, mephedrone, and gamma-hydroxybutyrate/gamma-butyrolactone (GHB/GBL) [1]. These substances are used alone or in combination and are taken orally, smoked, or injected. Injecting practices, referred to as “slamsex,” are especially concerning because they introduce additional risks of HIV and hepatitis C transmission through shared injection equipment [2].

Motivations for chemsex are varied. Studies describe its use to achieve disinhibition, escape stress, increase pleasure, and cope with stigma or negative emotions [3]. Chemsex differs from recreational drug use in that its primary goal is sexual enhancement. It often occurs in private settings and can last several days. Because chemsex sessions are often prolonged and involve multiple partners, they are frequently linked to condomless anal intercourse (CAI), transactional sex, and group sex [4], all of which are high-risk sexual behaviors that increase HIV transmission risk.

A pooled prevalence of chemsex among MSM was 19%, according to a systematic review of studies from Asian countries [5]. In the UK, nearly 30% of HIV-positive MSM reported participating in chemsex in the past year. Among this group, chemsex was strongly associated with unprotected anal sex, serodiscordant sex, bacterial STI diagnoses, and hepatitis C infection [6]. Bacterial STIs may increase susceptibility to HIV through mucosal inflammation, which further facilitates HIV transmission [7]. Clinic-based studies in London and Amsterdam also report higher rates of post-exposure prophylaxis use, HIV acquisition, and mental health issues among MSM who engage in chemsex [8]. Beyond increasing the risk of STIs, chemsex has been linked to adverse mental health outcomes. Anxiety, depression, psychosis, and cognitive impairment have been observed among men participating in extended sessions [8].

A meta-analysis of 23 Asian studies found that methamphetamine was the most commonly used substance during chemsex (pooled prevalence 16%), followed by GHB/GBL (15%) and ketamine (8%) [5]. These psychoactive substances may reduce inhibition and impair judgment, increasing engagement in behaviors associated with HIV acquisition and onward transmission [9]. Chemsex prevalence was significantly higher among MSM engaged in transactional sex and among those living with HIV [5]. These findings suggest that chemsex is an increasingly important HIV prevention challenge across Asia, particularly among populations already disproportionately affected by HIV infection. In response to these risks, comprehensive harm reduction strategies have become increasingly important for MSM who engage in chemsex. These approaches typically include access to pre-exposure prophylaxis (PrEP), regular HIV and STI screening, condom promotion, mental health support, and substance use services [1]. Although PrEP is highly effective in preventing HIV acquisition, it does not prevent other sexually transmitted infections or address harms associated with substance use [10].

In Malaysia, an online survey of 870 MSM found that 9% had engaged in chemsex in the past six months [11]. Participants who reported chemsex were more likely to have engaged in transactional sex, to know someone using PrEP, and to have been diagnosed with an STI in the past six months. Chemsex was also strongly associated with recent injection drug use [11]. In Vietnam, a socio-ecological study of MSM in Hanoi and Ho Chi Minh City found that 20% had used methamphetamine during sex, with 18% classified as engaging in high-risk use. Methamphetamine use was linked to selling sex behaviors, higher sexual sensation-seeking scores, and perceptions that chemsex was common within their networks [12]. These findings show how local social dynamics and drug availability can influence chemsex practices.

Qualitative studies in Singapore reveal similar patterns but also highlight local barriers. Participants reported using drugs to overcome fear of rejection and to enhance sexual pleasure. However, they also expressed serious concerns about strict drug laws and the criminalization of same-sex behavior, which deterred them from seeking help or sharing their chemsex experiences [13]. These structural issues, together with cultural stigma, may lead to underreporting and limit access to harm reduction services.

Although chemsex itself has not been studied in Myanmar, sexual risk behavior among MSM is well documented. A cross-sectional study of 256 MSM in Yangon and Mandalay found high rates of multiple partnerships and inconsistent condom use. More than 70% of participants reported having multiple partners in the past six months, and 56% of adult MSM and 61% of young MSM reported never or inconsistently using condoms with their permanent partners [14]. These findings indicate high-risk sexual behavior among MSM in Myanmar, which chemsex could amplify if present.

Myanmar’s sociopolitical environment further complicates this issue. Same-sex sexual activity remains criminalized under colonial-era laws, and drug use is heavily penalized, creating structural barriers to health-seeking behaviors and harm-reduction services [15]. Following the 2021 Myanmar military coup, political instability and conflict have severely disrupted Myanmar’s health system and HIV programs, further limiting access to prevention and care services [16]. At the same time, the widespread availability of illicit drugs and weakened governance increase access to psychoactive substances [17]. Persistent stigma from both society and healthcare providers continues to discourage MSM from accessing testing and prevention services. In the context of the ongoing HIV epidemic among MSM, limited harm-reduction services, and disruptions to HIV prevention programs following the 2021 military coup, chemsex may represent an emerging but unrecognized challenge for HIV prevention in Myanmar. However, chemsex practices remain largely undocumented in Myanmar.

Although international and regional research on chemsex is growing, no studies have specifically examined chemsex among MSM in Myanmar. Given the well-documented high-risk sexual behaviors, the widespread availability of psychoactive substances, and the structural vulnerabilities affecting MSM in Myanmar, understanding the extent and patterns of chemsex is important for HIV prevention and public health planning. Therefore, this study was conducted to assess the prevalence, practices, and factors associated with chemsex use among MSM in Yangon, Myanmar.

## Materials and methods

### Study design, area, and population

A cross-sectional online assessment was conducted using a quantitative research design from December 1, 2024, to January 25, 2025. Inclusion criteria included MSM aged 18 or older who live in Yangon, can read Burmese, and have had sexual experiences (with men or women) in the past six months.

### Operational definitions

Chemsex use (sexualized drug use) is defined as intentionally using psychoactive substances before or during sexual activity to prolong or intensify the experience, diversify sexual practices, or enhance sexual performance [18]. This study will focus on commonly used chemsex drugs in Myanmar, including cannabis, ecstasy, poppers, cocaine, and methamphetamine/ice.

Men who have Sex with Men (MSM) in this study are men who engage in sexual activity with other men, regardless of sexual orientation [19]. This group includes gay and bisexual men in Yangon, Myanmar.

High-risk sexual behaviors include unprotected sexual intercourse (oral or anal), multiple sexual partners, high-risk partners, and aggressive sexual acts such as fisting, which can cause abrasions and other physical injuries [20].

### Sample size and sampling

The sample size was calculated using a single-proportion formula. Because no prior studies on chemsex among MSM in Myanmar were available, a prevalence estimate of 19% from a recent systematic review and meta-analysis of studies among MSM in Asia was used [5]. Assuming a 95% confidence level, a 5% precision error, and a 10% allowance for missing data, the minimum required sample size was 261 participants.


$$\begin{array}{c} \text{N}={\text{Z}}^{\text{2}}*\text{p}*\text{q}/{\text{d}}^{\text{2}} \end{array}$$

For estimating the infinite population proportion

Proportion (p) = 0.19, Error (d) = 0.05

Alpha (α) = 0.05, Z (0.975) = 1.96

### Recruitment

Online recruitment was conducted through trusted, closed social networks within the population. Information about the study and its objectives was shared on social media platforms to encourage participation. Questionnaires were then distributed through common social media channels, including Facebook pages for Yangon-specific MSM dating groups, private Telegram groups, and dating and social networking apps such as Grindr and HeeSay. Recruitment took place from December 2024 to January 2025.

### Data collection

A structured questionnaire was developed based on a literature review. Quantitative data were collected using this questionnaire, which was distributed online via the KoboCollect platform. The questionnaire included 45 questions across five sections: 1) Background characteristics of participants; 2) Lifestyle and sexual practices; 3) HIV related characteristics of participants; 4) Sexualized drug use (Chemsex) and associated sexual practices; and 5) Attitudes regarding chemsex.

The developed questionnaire, excluding the sociodemographic section, was reviewed and validated for content validity by two experts. The overall content validity index (CVI) was 0.96, with individual CVI values of 1.00 for relevance and 0.94 for clarity and conciseness.

A brief advertisement was created to explain the study’s goals, eligibility requirements, and the principles of anonymity and confidentiality. Eligible participants were directed to the questionnaire via a link or by scanning a QR code. At the start of the questionnaire, we informed participants that participation was voluntary, their responses would be kept confidential, and they could withdraw at any time. We also emphasized that they could stop the questionnaire at any time if they felt uncomfortable or did not want to continue. To prevent multiple submissions, the basic protection method in KoboCollect was used to block duplicate entries from the same user on the same browser and device.

### Data management and analysis

Excel data files were exported from KoboCollect. Data management and analysis were conducted in SPSS. Descriptive statistics were reported using appropriate measures, including the mean, median, frequency, and percentage. Bivariate analyses were conducted using the chi-square test, and a p-value <0.05 was considered statistically significant. Logistic regression was performed to identify predictors of chemsex. Variables with a p-value <0.2 in the bivariate analysis were included in the initial multivariable logistic regression model. Backward stepwise regression was then applied to obtain the final model.

### Ethical consideration

The objectives of the assessment were clearly stated at the start of the form, and all participants provided informed consent. Participant information was kept strictly anonymous and confidential. It was also made clear that participation in the study posed minimal risk and involved minimal intrusion into personal information. We emphasized that participants could withdraw at any time for any reason, such as feeling uncomfortable with the questions. To protect privacy and confidentiality, we did not ask for any personal identifiers, including email addresses. Data collected through the KoboCollect platform is secured, encrypted, and protected against unauthorized access. Only authorized research team members could access the data, and strict data-handling procedures were followed to ensure confidentiality. Additionally, participants were informed of these procedures and provided consent prior to data collection. Ethical approval was secured from the Ethics Review Committee of the Ministry of Health, National Unity Government, Myanmar (Ethics/NUG-MOH/2024/06).

## Results

### Demographic characteristics of participants

A total of 415 MSM were included in the study, with a mean age of 30.1 years (SD = 7.1; range 18–56 years). The majority (71.3%) were younger than 35 years. Most participants identified as gay (76.4%), and 23.6% identified as bisexual.

Regarding education, over three-fifths of respondents (62.2%) had attained a bachelor’s degree or higher, 21.4% were current university students, and 16.4% had completed only basic education. Employment was common among participants: 59% were employed, 29.2% were engaged in freelance or business activities, and 11.8% were unemployed. Only a small proportion (4.3%) reported working in entertainment-related occupations.

Monthly income varied, with 51.1% earning more than 600,001 MMK, 39.8% earning up to 600,000 MMK, and 9.2% reporting no income. Regarding living arrangements, 39.3% lived with their parents, 34.0% lived with others, and 26.7% lived alone.

### HIV related characteristics of participants

At the time of the survey, 14.2% of participants self-reported living with HIV, 71.3% reported being HIV-negative, 10.1% were unsure of their HIV status, and 4.3% declined to disclose their HIV status. Among participants living with HIV, 96.6% were receiving antiretroviral therapy (ART). Current use of PrEP was reported by 29.1% of participants.

### Prevalence of chemsex

Overall, 26.0% of participants reported engaging in chemsex at some point, while 74.0% had never participated. Among those who had, the mean age at first chemsex experience was 26.7 years (SD = 6.2; range 15–44 years). In the six months before the survey, 20.7% reported engaging in chemsex, whereas 5.3% reported prior experience but not within the past six months.

### Behaviors and sexual practices during chemsex

**Fig 1:**
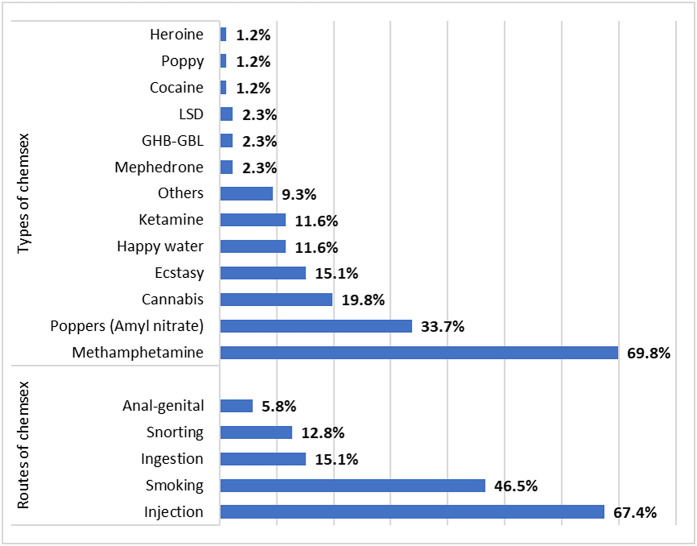
Types and routes of chemsex.

Among the 108 participants who reported chemsex, methamphetamine was the most commonly used substance (69.8%). Drug use was predominantly via injection (67.4%) (Fig 1), with over half self-injecting (62.1%) or being injected by others (67.2%). Sharing injection equipment was relatively rare (8.6%). Chemsex most commonly occurred in hotels (76.7%) and private homes (54.7%). Nearly half (46.5%) of the sessions lasted between 4 and 12 hours, and 41.9% lasted less than 4 hours.

Condom use during chemsex in the past six months was inconsistent, with only 10.5% always using condoms, 52.3% sometimes, and 24.4% never. Group sex during chemsex was reported by 40.7%, usually involving threesomes (57.1%) or groups of 4–5 (40%). Condom use during group sex was also inconsistent, with only 11.4% always using condoms, 54.3% sometimes, and 22.9% never.

Regarding the most recent chemsex, 46.5% reported chemsex within the past two weeks, 14.0% within the past month, and 23.3% within the past three months.

Adverse outcomes linked to chemsex were common. Over half (52.3%) experienced unpleasant physical sensations, 38.4% experienced anxiety or panic attacks, 22.1% experienced paranoia, and 19.8% exhibited irritability or aggressiveness. A small portion reported overdose with loss of consciousness (3.5%), suicidal thoughts (2.3%), or suicide attempts (2.3%). One in ten (10.5%) missed regular medication because of chemsex, while 30.2% were unsure. Additionally, 30.2% used illicit drugs outside sexual contexts.

### Attitude regarding chemsex

**Fig 2:**
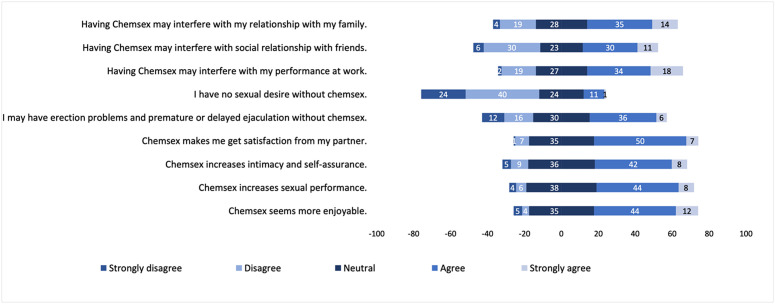
Attitude regarding chemsex.

Participants held mixed views on chemsex, with a substantial proportion recognizing benefits alongside notable concerns. Nearly half reported that chemsex made sexual experiences more enjoyable (44%) and enhanced performance (44%), and many also reported increased intimacy (42%) and greater satisfaction with their partner (50%). However, dependence-related issues were acknowledged, as 36% indicated potential erection or ejaculation problems without chemsex, though most disagreed with a complete loss of desire. Negative impacts were also evident, with approximately one-third of respondents reporting interference with their work (34%), family relationships (35%), and social interactions (30%) (Fig 2).

### Associated factors of chemsex

Bivariate analysis found no significant associations between chemsex use and age, education, or living status, although certain behavioral and identity-related factors were influential. Gay-identifying MSM were significantly more likely to engage in chemsex than bisexual men (29.7% vs. 14.3%, p = 0.002). Smoking status was strongly associated, with both current (35.2%) and former smokers (34.5%) reporting higher chemsex use than never-smokers (17.3%, p < 0.001). Alcohol use showed a trend toward higher prevalence with increased drinking frequency, though this did not reach statistical significance (p = 0.05) (Table 1).

**Table 1:** Bivariate analysis of socio-demographic characteristics and chemsex.

Characteristic	Chemsex use (n, %)		Total	P value
	No	Yes		
Age				
18-34	215 (72.5)	81 (27.4)	296 (100.0)	0.326
> 34	92 (77.3)	27 (22.7)	119 (100.0)	
Type of MSM				
Gay	223(70.3)	94 (29.7)	317 (100.0)	0.002
Bisexual	84 (85.7)	14 (14.3)	98 (100.0)	
Highest education				
Basic	53 (77.9)	15 (22.1)	68 (100.0)	0.521
University	68 (76.4)	21 (23.6)	89 (100.0)	
Bachelor & Post- graduate	186 (72.1)	72 (27.9)	258 (100.0)	
Living status				
Alone	81 (73)	30 (27)	111 (100.0)	0.778
With others	226 (74.3)	78 (25.7)	304 (100.0)	
Smoking				
Never smoked	172 (82.7)	36 (17.3)	208 (100.0)	<0.001
Ex-smoker	78 (65.5)	41 (34.5)	119 (100.0)	
Current smoker	57 (64.8)	31 (35.2)	88 (100.0)	
Alcohol				
Never	76 (83.5)	15 (16.5)	91 (100.0)	0.054
Up to 3 times a month	190 (72)	74 (28)	264 (100.0)	
4 times and above a month	41 (68.3)	19 (31.7)	60 (100.0)	

Engagement in transactional sex was also associated with chemsex use. Those offering sexual services for money reported higher chemsex use (47.4% vs. 25.0%, p = 0.03), and those paying for sex showed a borderline association (36.2% vs. 24.4%, p = 0.05). Furthermore, dating app use was significantly associated with higher chemsex involvement compared with non-users (28.1% vs. 8.9%, p = 0.006) (Table 2).

**Table 2:** Bivariate analysis of sexual practices and chemsex.

Characteristic	Chemsex use (n, %)		Total	P value
	No	Yes		
Regular partner				
No	198 (76.2)	62 (23.8)	260 (100.0)	0.190
Yes	109 (70.3)	46 (29.7)	155 (100.0)	
Sexual Practices				
Receptive	166 (72.2)	64 (27.8)	230 (100.0)	0.351
Insertive	166 (70.9)	68 (29.1)	234 (100.0)	0.109
Oral	141 (71.9)	55 (28.1)	196 (100.0)	0.371
Fisting	58 (73.4)	21 (26.6)	79 (100.0)	0.900
Sex toys	19 (70.4)	8 (29.6)	27 (100.0)	0.659
Others	37 (69.8)	16 (30.2)	53 (100.0)	0.459
Offered sexual services for money				
No	297 (75)	99 (25)	396 (100.0)	0.030
Yes	10 (52.6)	9 (47.4)	19 (100.0)	
Money paid for sexual service				
No	270 (75.6)	87 (24.4)	357 (100.0)	0.057
Yes	37 (63.8)	21 (36.2)	58 (100.0)	
Using dating apps				
No	41 (91.1)	4 (8.9)	45 (100.0)	0.006
Yes	266 (71.9)	104 (28.1)	370 (100.0)	

Multivariate analysis identified several independent predictors of chemsex use among MSM. Gay-identifying participants were three times more likely to engage in chemsex than bisexual men (AOR 3.0, 95% CI 1.5–5.9, p = 0.001). Smoking was also a significant factor, with smokers more than twice as likely to use chemsex (AOR 2.3, 95% CI 1.2–4.4, p = 0.006). Sexual behaviors showed the strongest associations: participation in group sex increased the likelihood fourfold (AOR 4.0, 95% CI 2.4–6.6, p < 0.001), and having multiple partners tripled the odds (AOR 3.3, 95% CI 1.7–6.3, p < 0.001) (Table 3). These findings highlight identity- and behavior-related risk factors as key determinants of chemsex engagement.

**Table 3:** Multivariate analysis on associated factors of chemsex.

Characteristics^	Crude OR (95% CI)	Adjusted OR (95% CI)	P value
Type of MSM*			0.001
Bisexual	ref.	ref.	
Gay	2.5 (1.3, 4.6)	3.0 (1.5, 5.9)	
Smoking*			0.006
Never	ref.	ref.	
Ex-smoker	2.5 (1.5, 4.2)	2.3 (1.3, 4.1)	
Current smoker	2.6 (1.5, 4.6)	2.3 (1.2, 4.4)	
Alcohol			0.817
Never	ref.	ref.	
Up to 3 times a month	2.0 (1.1, 3.7)	1.3 (0.6, 2.6)	
4 times and more a month	2.3 (1.1, 5.1)	1.1 (0.4, 3.6)	
Money paid for sexual service			0.211
No	ref.	ref.	
Yes	1.8 (0.8, 3.2)	1.6 (0.8, 3.1)	
Offered sexual services for money			0.643
No	ref.	ref.	
Yes	2.7 (1.1, 6.8)	1.3 (0.5, 3.6)	
Using dating apps			0.185
No	ref.	ref.	
Yes	4.0 (1.4, 11.5)	2.2 (0.7, 6.9)	
Group sex*			<0.001
No	ref.	ref.	
Yes	5.6 (3.5, 9.0)	4.0 (2.4, 6.6)	
Multiple Partners*			<0.001
No	ref.	ref.	
Yes	5.3 (2.9, 9.7)	3.3 (1.7, 6.3)	

^ Eight variables were included in the first logistic regression model; *Four predictor variables remained in the final logistic regression model.

## Discussion

This study highlights the growing importance of chemsex within the MSM community in Yangon, Myanmar, which calls for urgent intervention to promote harm reduction and education in the country. We found that more than one-quarter of participants had engaged in chemsex at some point, and one in five reported doing chemsex in the last six months. These findings exceed those reported in several neighboring countries. For instance, in Malaysia, a national survey indicated a prevalence of 9% [11], while a systematic review of Asian data showed estimates ranging from 3% to 29%, depending on the population and context [5]. The relatively high prevalence in our study may be linked to easy access to methamphetamine in Myanmar and the concentration of younger, urban MSM in Yangon who are more exposed to digital networks and peer influences.

The current study revealed that methamphetamine was the most commonly used substance (69.8%), with injection as the main route of administration (67.4%). This is concerning because injecting drug use, or “slam sex,” is associated with very high risks of HIV and hepatitis C transmission through needle sharing and mucosal trauma [2]. However, needle sharing was relatively rare in our study (8.6%). Still, international evidence shows that even minimal sharing can sustain transmission of blood-borne infections within high-prevalence sexual networks [6].

Chemsex was strongly linked to behavioral risks. Our study found that group sex was four times more common during chemsex, and having multiple partners was three times more frequent. Condom use during chemsex was inconsistent, with more than half reporting they sometimes used condoms and one-quarter never using them. These findings are similar to those among HIV-positive MSM in the UK, where chemsex was associated with unprotected anal sex, serodiscordant sex with a detectable viral load, and STI acquisition [4]. Similarly, in Spain, chemsex was independently associated with higher prevalence of STIs and hepatitis C [6]. In Myanmar, MSM already report high baseline levels of condomless sex and multiple partners [14], suggesting that chemsex may intensify existing risks.

The relatively low prevalence of needle sharing, compared with the high prevalence of sexual risk behaviors, suggests that sexual transmission may be a more important route of HIV transmission than injection-related transmission among MSM engaging in chemsex in Yangon. The combination of inconsistent condom use, multiple sexual partners, and group sex creates conditions that facilitate HIV and STI transmission within interconnected sexual networks. These factors should be prioritized in prevention programs.

Mental health consequences were also reported among our study participants. Nearly 40% of participants experienced anxiety or panic attacks, while smaller groups reported paranoia, suicidal thoughts, or attempts. These results align with evidence from Europe and Asia showing chemsex-related anxiety, depression, and psychiatric admissions [5,18]. Notably, a tenth of participants reported missing regular medication due to chemsex, highlighting the risks of ART non-adherence among people living with HIV.

Our findings also highlight the influence of identity and digital platforms on chemsex behaviors. Gay-identifying MSM were three times more likely than bisexual men to engage in chemsex, a pattern also observed in neighboring Malaysia and Singapore [11,13]. Nearly nine out of ten participants used dating apps, and those who used them were significantly more likely to participate in chemsex. This demonstrates that geosocial networking platforms have become crucial for organizing sexualized drug use across Asia [8].

The HIV related characteristics observed in this study provide further insight into opportunities for HIV prevention among MSM engaging in chemsex. Approximately one in seven participants self-reported living with HIV, and nearly all were receiving antiretroviral therapy, indicating strong engagement with HIV treatment services. Despite current PrEP use among nearly one-third of participants, high-risk sexual behaviors remained common among those engaging in chemsex. This highlights the need for comprehensive HIV prevention approaches that integrate PrEP, behavioral interventions, and harm reduction.

Several limitations should be acknowledged. First, the cross-sectional design limits the ability to establish causal relationships between chemsex and associated risk factors. Second, self-reported data on sensitive behaviors may be subject to recall bias. Although the anonymous survey format may have reduced underreporting, some participants may still have been reluctant to disclose stigmatized behaviors. Third, participants were recruited through social media platforms and geosocial networking applications commonly used by MSM. Consequently, MSM with limited internet access, lower digital literacy, or those who do not use these platforms may have been excluded from the study. Participants recruited may also have been younger, more educated, more urban, and more connected to MSM social networks than the broader MSM population. Therefore, selection bias may have occurred, and the findings may not be fully representative of all MSM in Yangon or Myanmar, particularly those who are older, more hidden, or living in rural areas. The generalizability of the findings should therefore be interpreted with caution. Comparisons with studies using different recruitment methods should also be made carefully.

In summary, this study demonstrates that chemsex is a significant and growing public health concern among MSM in Yangon. Chemsex was reported by one in four MSM, and one in five had engaged in it recently. Although most participants were young and well educated, chemsex was primarily linked to behavioral factors rather than basic demographics. Methamphetamine use, predominantly by injection, was common, and condom use during chemsex and group sex was inconsistent, increasing sexual health risks. Many participants reported negative physical and psychological effects, even though some perceived greater sexual pleasure and intimacy. Gay identity, smoking, group sex, and having multiple partners were key factors independently associated with chemsex.

The findings highlight the urgent need for policy and intervention actions. First, harm reduction services for sterile injection equipment, overdose prevention, and safer drug use education are essential. Second, HIV prevention strategies should prioritize sexual risk reduction in chemsex settings. PrEP should be integrated with regular HIV and STI testing, condom promotion, sexual health education, substance use support, and mental health services. This integrated approach acknowledges that chemsex involves both sexual and substance use behaviors and requires coordinated HIV prevention, harm reduction, and community-based support. Third, digital outreach through dating apps and social media should be used to promote health messages and connect MSM to services, recognizing the significance of online platforms in chemsex participation. Fourth, reducing stigma and discrimination is vital to improving disclosure and access to services.

Future studies should use longitudinal designs to examine causal relationships among chemsex, sexual risk behaviors, and health outcomes. Incorporating testing for HIV, STIs, and hepatitis C will help validate self-reported data and improve assessments of transmission risk. Qualitative research is also needed to understand the lived experiences of MSM in Myanmar, including their motivations for chemsex, coping mechanisms, and barriers to care. Finally, intervention studies are crucial for assessing the effectiveness of community-based harm reduction, digital outreach, and integrated mental health and sexual health services tailored to MSM who engage in chemsex.

## Supporting information

S1 Fig Types and routes of chemsex. (TIF)

S2 Fig Attitude regarding chemsex. (TIF)

S1 Table Bivariate analysis of socio-demographic characteristics and chemsex. (TIF)

S2 Table Bivariate analysis of sexual practices and chemsex. (TIF)

S3 Table Multivariate analysis on associated factors of chemsex. ^ Eight variables were put in the first model of logistic regression; *Four predictor variables remained in the final logistic regression model.
(TIF)

S1 Data Dataset for Chemsex among MSM in Yangon, Myanmar. https://doi.org/10.6084/m9.figshare.32736654.
(XLSX)
